# A COLLABORATIVE PILOT FOR AGE-FRIENDLY HOSPITAL CARE

**DOI:** 10.1093/geroni/igad104.0194

**Published:** 2023-12-21

**Authors:** Kari Hirvela

**Affiliations:** UW Health, Madison, Wisconsin, United States

## Abstract

To ensure older adults receive the best care possible, Age Friendly Health Systems has become a national effort in the US. This initiative is based on 4 core components (called 4 M’s framework), What Matters, Medication, Mentation, and Mobility. We piloted a multi-disciplinary care model based on the 4 M’s framework to improve healthcare delivery for older adults at a large academic hospital. Using the Plan-Do-Check-Act, a care model was developed. Plan: A multi-disciplinary stakeholder group identified best practices for each of the 4Ms, including: 1) asking and documenting “what Matters most” and mobility; 2) pharmacy review of high-risk medications; 3) creation of 4M provider patient list in electronic health record (EHR); 4) implementation of delirium rounds. Education was provided to hospital champions. An interactive dashboard was created to demonstrate impact. Do: The 4M’s were implemented for patients ≥ 65 years old on 2 inpatient units. Check: 4M’s interventions was monitored via dashboard. Findings revealed low provider utilization of the patient list template and provider-initiated adaptations to the care plan. Feedback hospital providers was largely positive. Act: Based on feedback, revisions were made within the EHR, and a 4M’s patient flyer was created. 73% of older adults received 4M’s care on the 2 pilot units, meeting the criteria demonstrating “Committed to Care Excellence” by the Institute of Healthcare Improvement. We were able to effective implement 4 M’s care. Age friendly care is an evidence-based model that may improve outcomes for older adults.

